# Determining the caregivers’ burden in caregivers of patients with mental illness

**DOI:** 10.12669/pjms.35.5.720

**Published:** 2019

**Authors:** Salma Siddiqui, Javeria Khalid

**Affiliations:** 1 Salma Siddiqui, Department of Behavioral Sciences, National University of Sciences & Technology, Islamabad, Pakistan; 2 Javeria Khalid, MS Scholar, Department of Behavioral Sciences, National University of Sciences & Technology, Islamabad, Pakistan

**Keywords:** Burden of Illness, Caregiver, Coping Behavior, Family caregivers, Informal Care, Psychiatric Patients

## Abstract

**Objective:**

To examine the factors associated with caregivers’ burden in individuals providing care to family members suffering from serious mental illness.

**Methods:**

This Cross Sectional Study was carried out at Armed Forces Institute of Mental Health, Rawalpindi, from May 2015 to December 2015. A purposive sample of 120 family caregivers (60 males and 60 females, age range= 18-65) who were taking care of patients with serious mental illness (i.e. Major Depressive Disorder, Bipolar Disorder & Schizophrenia) for at least one year were recruited from the hospital and assessed through Zarit Burden Interview (ZBI) and Brief COPE inventory. The decline in functional status, and diminished physical capacity compromising the independent living of the care recipient was assessed through Katz Index of Independence in Activities of daily living (ADL) and Lawton Instrumental activities of daily living (IADL).

**Results:**

The results suggest that the longer the duration of illness (F=25.71, p < 0.01), with increased impairments of care-recipients, (decline in functional status, F=21.33, p < 0.001; diminished physical capacity F =32.41, p < 0.001) the more the burden experienced by the caregivers. Moreover, caregivers who were married (t=-2.98, p < .01), less educated (t =5.48, p < .01), lived in rural area (t = -7.99, p < .01), had lower monthly income (t = -4.95, p < .01) provide longer hours of caregiving (F=19.12, p < 0.001) and used avoidant coping behavior (F= 56.37, p < 0.001) reported significantly higher caregiver burden than caregivers who were unmarried, more educated, lived in urban area and had better income.

**Conclusion:**

The results of study demonstrate that caring for family members with serious mental illness impacts the caregivers’ wellbeing. It, therefore, highlights the need for support and counseling services for the caregivers to reduce the burden of caring.

## INTRODUCTION

Providing care to family members suffering from prolonged illness challenges the wellbeing of the caregivers. The task of caregiving becomes further challenging if the family member is suffering from a serious mental illness as the stigma of mental illness adds to this burden of caring. This assumes a critical significance as the prevalence of mental illness is gradually increasing with approximately 151 million people suffering from depression and 26 million from schizophrenia (World Health Organization, 2010).^1^ There is also a steady increase of mental illness cases in Asian region. Though the epidemiological researches in the context of Pakistan are scarce, however, from the available data we can infer that the situation would be similar here too.

The patients suffering with serious mental illness develop a strong dependency on caregivers mainly due to the significant impairment associated with their illness. This dependency and responsibility for caring affect quality of life of caregivers impacting their health, work, socializing, relationships and adds to their distress.^2^ The literature differentiates burden with regard to its impact as objective (e.g. disruption in caregiver’s life in terms of household routine, social activities and financial/employment difficulties) and subjective (emotional distress experienced by caregivers, e.g. sadness, fear, anger, guilt, loss, stigma and rejection.^3^) Moreover, the socio-demographical factors and illness related aspects of both care-recipients and caregivers have also been seen as adding to care giver’s burden.^4^,^5^ This may result in a neglect of caregivers’ own health and other social and personal needs, which in turn reduces their capacity to deal with the care demands efficiently.^6^

Care givers’ burden is slowly being recognized in Pakistan, especially in the context of chronic conditions^7^ and with regard to mental illness as well.^8^-^10^ As the caregiving in Pakistan mostly depends on the informal caregiving by the close family members^11^, it is important that its impact is examined. It would be pertinent to determine the factors which contribute to the caregivers’ burden. The current study, therefore, examines the factors which explain the burden experienced by caregivers due to caring for family member suffering from mental illness. These factors are sociodemographic aspects of both the caregivers and care recipients; functional state of the mentally ill family member and the coping behavior used by the caregivers. It also aims to determine the predictors of caregivers’ burden. It is contended that identifying the factors associated with burden will inform us about the relevant preventive measures and suggest timely intervention to prevent the debilitating effect of the caregiver’s burden.

## METHODS

The study included 120 family members (60 males & 60 females) who were primary care givers, for at least one year, of patients suffering from schizophrenia, bipolar disorder or Major depressive disorders without any comorbid illness. They were approached from both outpatient and inpatient departments of Armed Forces Institute Mental Health (AFIMH) Rawalpindi.

Care recipient’s physical health status were assessed through Katz Index of Independence in Activities of daily living (ADL)^12^ and Lawton Instrumental activities of daily living (IADL)^13^ which assess functional status and capacity to perform everyday tasks. The care giver’s stress was assessed through Zarit Burden Interview^14^ (ZBI, 22 items, translated in Urdu alpha reliability = 0.91) and their coping behaviors was assessed through Brief COPE Inventory^15^ (28 items, translated in Urdu, alpha reliability = 0.71). Details of demographic information was obtained through a questionnaire constructed for the study. The study ensured the ethical parameters indicated for human subject research and the permission to access to patients from AFIMH was obtained. The participants were ensured about the anonymity, confidentiality and right to withdraw from research. After obtaining informed consent, each participant was individually interviewed and information was obtained through the questionnaires. During data collection, if any participant showed signs of distress, they were either provided brief counseling with guidance to ensure self-care in the daily life or where required a referral to the available consultant Psychiatrist was made.

## RESULTS

The data was first checked for normality and accuracy then analyses were carried out with the help of SPSS (version 18). The skewness of the data was found to be in the accepted range (1.96, p< .05). The statistical analyses were carried out to determine mean differences through t-test and ANOVA and prediction analysis were carried out through regression analysis after analyzing the association through Pearson’s and biserial correlations between various factors and outcome variable.

The mean age of care-recipients was 34.6 years (SD = 12.3), and predominantly women (61%) who had serious mental illness (major depressive disorder (46%), bipolar disorder (42%) and schizophrenia (32%). They included parents (25%), children (30%), siblings (25%), and spouses (24%).

The caregivers who were married (t=-2.98, p < .01), less educated (t =5.48, p < .01), lived in rural area (t = -7.99, p < .01), and had lower monthly income (t = -4.95, p < .01) experienced significantly higher caregivers’ burden than caregivers who were unmarried, more educated, lived in urban area and had better income.

The analysis of increasing levels of burden (Table-I), show that the longer the duration of illness (F=25.71, p < 0.01), with increased impairments of care-recipients (i.e. decline in functional status, F=21.33, p < 0.001; diminished physical capacity F =32.41, p < 0.001) the more the burden experienced by the caregivers. Moreover, caregivers providing longer hours of caregiving (F=19.12, p < 0.001) and using avoidant coping (F= 56.37, p < 0.001) reported higher level of burden.

**Table I:** Comparison of Means (ANOVA) for Illness and Caregiving-Related Factors and Caregivers’ Burden (N = 120).

Variable	Total	Mild to moderate burden (n = 41)	Moderate to severe burden (n = 60)	Severe burden (n = 19)	

		M (SD)	M (SD)	M (SD)	F
ADLs		17.4 (2.5)	24.3 (3.6)	29.1 (3.1)	21.33**
IADLs		19.2 (2.1)	26.7 (2.4)	32.3 (3.5)	32.41**
Duration of illness		15.1 (2.2)	21.1 (1.8)	27.1 (2.7)	25.71**
Hours of caregiving/week		19.3 (2.2)	22.5 (2.8)	26.3 (3.1)	19.12**
Problem-focused coping		27.1 (1.6)	21.0 (3.1)	17.5 (0.5)	62.54***
Emotion-focused coping		35.1 (3.2)	30.8 (3.9)	28.2 (1.9)	16.57***
Avoidant coping		13.5 (2.2)	19.9 (3.8)	27.0 (3.3)	56.37***

** ***Note.*** p < .01,

*** p < .001. ADL, activities of daily living; IADL, instrumental activities of daily living;

Moreover, except for caregivers’ age and gender, moderate to strong significant relationships exists between caregiver‘s socio-demographical factors (caregivers’ marital status, education, residence and income); illness-related factors (functional status of patients and duration of their illness), caregiving-related factors (hours of caregiving and coping behavior of caregivers) and caregivers’ burden (Table-II). The hierarchical multiple regression (Table-III) examined the predictive relationships of variables for caregivers’ burden. The overall model with all eleven predictors was statistically significant and explained 45% of the variance for caregivers’ burden (F (11, 71) = 21.28, p < 0.01). In Block 1, caregivers’ marital status, education, residence area and income explained 16% of the variance for caregivers’ burden, (F (4, 78) = 12.08, p < 0.01). In Block 2 (controlling socio-demographical factors of Block 1), the decline in functional status, diminished physical capacity and duration of illness of the care recipients explained a statistically significant variance (17%) for caregivers’ burden, (F (3, 75) = 31.50, p < 0.01). In Block 3, controlling all the other variables, the number of hours of caregiving and coping behaviors explained 12% of variance of caregivers’ burden, (4, 71= 16.29, p < 0.01). Therefore, all three blocks of variables appear to contribute significantly in the prediction of caregivers’ burden.

**Table II:** Associations between Predictor Variables and Caregivers’ burden Pearson’s and biserial correlations for the association of care-recipients’ and caregivers’ demographical, illness and caregiving-related factors with caregivers’ burden (N = 120).

Variable	Caregiver burden	1	2	3	4	5	6	7	8	9	10	11
1. Caregiver marital status #	0.30**	-	-0.52**	0.27*	-0.27*	0.05	0.07	-0.04	-0.18	-0.30**	-0.17	0.23*
2. Caregiver education	-0.53**	-	-	-0.55**	0.51**	-0.04	-0.06	-0.10	0.12	0.41*	0.25*	-0.39*
3. Residence area #	0.67**	-	-	-	-0.65**	0.20	0.22	0.22*	0.13	-0.68**	-0.63*	0.73**
4. Household income	-0.66**	-	-	-	-	-0.21	-0.18	-0.06	-0.17	0.64*	0.43*	-0.57*
5. ADLs	0.27**	-	-	-	-	-	0.33*	0.35*	0.32*	-0.24*	-0.21*	0.22*
6. IADLs	0.46**	-	-		-	-	-	0.45*	0.50*	-0.27*	-0.23*	0.25*
7. Duration of illness	0.23*	-	-	-	-	-	-	-	0.29*	-0.26*	-0.18	0.28*
8. hours of caregiving/week	0.50**	-	-		-	-	-	-	-	0.12	0.09	0.06
9. Problem-focused coping	-0.86**	-	-		-	-	-	-	-	-	0.63*	-0.86*
10. Emotion-focused coping	-0.61**	-	-		-	-	-	-	-	-	-	-0.66*
11. Avoidant coping	0.79**	-	-		-	-	-	-	-	-	-	-

***Note:*** # biserial correlation;

* p < .05,

** p < .01, ***p < .001; ADL, activities of daily living; IADL, instrumental activities of daily living.

**Table III:** Analysis of Multidimensional factors in Predicting Caregivers’ Burden Hierarchical multiple regression analysis (N = 120).

	R^2^ change	F ratio for R^2^ change	B	SE	Β
Caregivers’ marital status			0.23*	0.03	0.21
Caregivers’ education	0.16	12.08**	0.30*	0.14	-0.13
Caregivers’ residence area			0.32*	0.15	0.23
Caregivers’ income			0.68*	0.32	-0.12
ADLs			0.21**	0.06	0.18
IADLs	0.17	31.50**	0.21**	0.03	0.34
Duration of illness			0.24**	0.09	0.30
Number of hours of caregiving/week			0.63**	0.13	0.32
Problem-focused coping	0.12	16.29**	0.17**	0.03	-0.27
Emotion-focused coping			0.15*	0.06	-0.24
Avoidant coping			0.21**	0.02	0.29
R^2^			0..45**		

* ***Note:*** p < .05,

** p < .01, ***p < .001; ADL, activities of daily living; IADL, instrumental activities of daily living.

## DISCUSSION

The study examined the contribution of socio-demographic and illness related factors to the burden experienced by caregivers of patients with serious mental health conditions. The findings suggest that all three groups of predictors (caregiver socio-demographical factors, illness-related factors and caregiving-related variables) explained caregivers’ burden. The illness-related factors, indicating functional status of care-recipients, were the most significant predictors in the study, contributing to 17% of caregiver burden, followed by caregivers’ socio-demographical and caregiving-related factors, each of which represented 16% and 12% of caregivers’ burden respectively. The findings highlight that caring for family members with serious mental health conditions compromises the wellbeing of the caregivers and the risk increases for caregivers who live in a rural area, have minimum education, meager income and have limited resources to engage in problem solving. It also highlights that with increased dependency on caregivers, more hours of caring would be expected from the caregivers; which would exhaust the caregivers’ capacity to manage care demands with problem solving style. This may lead to resorting to emotion-focused strategies, i.e. practices based on religion or social connections such as venting or sharing and religious practices and rituals. This may also result in caregiver’s fatigue leading to avoidance of responsibility. It appears plausible that the demands of caring, along with caregivers own emotional load would reduce their capacity to focus the care demand in a pragmatic manner. The distress experienced due to increasing care needs of the mentally ill family member thus compromises the quality of life of the caregivers. The care recipient being a close family member, cost and stigma of mental illness,^16^ therefore, results in reluctance to take help from other formal or informal care givers.

The study highlights the need to counsel the family caregivers and facilitate them when they accompany their family members for their health needs. This is important to ensure the sustained care received by the mentally ill patients and help the caregivers’ stay in good health themselves to take care of the care demands. Furthermore, as many socio demographic factors- for instance income, education, residence in rural areas- contribute to caregiver’s burden, a formal care giving service needs to be established which is regulated by government. This is a neglected area in Pakistan and requires the attention of all stakeholders. Besides making the formal caregiving service available, the approach to community wellness and mental health^17^, which encourages self-care and healthy coping behaviors, requires attention of both health service providers and academia. This would help provide support to caregivers without stigmatizing their emotional distress and help prevent, manage and improve the quality of life of caregivers. The health professionals also need to be educated to pay attention to the counseling needs and informational care of individuals caring for family members with serious mental health conditions.

### Limitations

The study though highlights the role of socio demographic and illness related factors as contributing to caregivers’ burden, the sample was limited to one tertiary care setting where most of the patients were entitled for treatment because of either having served in Armed Forces or having a family member serving in Army. It is expected that level of burden would change if the cost to treatment is also involved as other associated sociodemographic factors are seen in this study to increase the burden. It would, therefore, be of interest to carry out further research which rectifies this limitation.

## CONCLUSION

A moderate to severe level of caregivers’ burden was found among individuals caring for family members suffering from serious mental illness. Functional decline of care-recipients followed by caregivers’ socio-demographical factors and caregiving-related factors were found to be the most significant predictors of caregivers’ burden.

### Author’s Contribution

**SS:** Contributed to research design and analysis, supervised the research, sought approval from the Study Site and Ethical board. Improved and updated the write up for publication.

**JK:** Designing, Data Collection, Statistical data analysis and Interpretation of data, preparation of the manuscript.
